# An Innovative Selective Fluorescence Sensor for Quantification of Hazardous Food Colorant Allura Red in Beverages Using Nitrogen-Doped Carbon Quantum Dots

**DOI:** 10.1007/s10895-023-03303-2

**Published:** 2023-06-17

**Authors:** Baher I. Salman

**Affiliations:** Pharmaceutical Analytical Chemistry Department, Faculty of Pharmacy, Al-Azhar University, Assiut branch, Assiut, 71524 Egypt

**Keywords:** AR, N@CQDs, Beverages, Ion Pair, Fluorescence

## Abstract

An innovative simple, sensitive, and selective method has been developed and validated for quantification of hazardous Allura red (**AR, E129**) dye in beverages. Allura red (AR) is a synthetic dye that is commonly used in the food industry to give foods a bright and appealing color. The method is based on microwave-assistant nitrogen-doped carbon quantum dots (N@CQDs) from a very cheap source with a high quantum yield equal to (36.60%). The mechanism of the reaction is based on an ion-pair association complex between AR and nitrogen-doped carbon quantum dots (N@CQDs) at pH 3.2. The reaction between AR and N@CQDs led to a quenching effect of the fluorescence intensity of N@CQDs at 445 nm after excitation at 350 nm. Moreover, the quantum method’s linearity covered the range between 0.07 and 10.0 µg mL^− 1^ with a regression coefficient is 0.9992. The presented work has been validated by ICH criteria. High-resolution transmission electron microscopy (HR-TEM), X-ray photon spectroscopy (XPS), Zeta potential measurements, fluorescence, UV-VIS, and FTIR spectroscopy have all been used to fully characterize of the N@CQDs. The N@CQDs were successfully utilized in different applications (beverages) with high accuracy.

**Supplementary Information:**

The online version contains supplementary material available at 10.1007/s10895-023-03303-2.

## Introduction

Allura red (**AR**), one of the popular colorant food azo dyes, is a vibrant red shade often used in a variety of food with E number (E 129) and beverage products [1]. It adds a significant application as color to candies, beverages, ice cream, sodas, and many other delicacies. Despite concerns raised by some researchers over its safety, AR remains one of the most widely used food dyes. Many experts attest to its safety, stating that it is regulated by food safety agencies and poses no harm to human health when consumed in appropriate amounts as an allergic effect. Despite this, some individuals may have sensitivity to AR and may experience mild allergic reactions [1–3]. Therefore, it is always essential to read food labels and consult a healthcare professional if you have any concerns about the ingredients.

Allura red is (Figure S1, Supplementary materials) 6-hydroxy-5-[(2-methoxy-5-methyl-4-sulfonatophenyl) diazenyl]naphthalene-2-sulfonate disodium salt.

Different analytical approaches were utilized for AR analysis as spectrophotometric [4–8], fluorimetric [9], electrochemical [10–13], and HPLC methods [14, 15].

Determination of Allura red is a crucial task in the food industry as it is extensively used as a colorant. However, conventional methods for its detection are time-consuming, lack selectivity, low sensitivity, using organic solvents, need pretreatment of the samples before analysis and require expensive equipments as observed in the reported methods [4–15]. The presented work involves reaction of N@CQDs with AR and monitoring changes in fluorescence intensity using a spectrofluorometer approach. The results can be obtained within minutes without any need for complex instrumentation or skilled technicians. Furthermore, N@CQDs can detect very low concentrations of AR accurately even in complex matrices such as soft drinks. Therefore, this novel approach holds great promise for the rapid screening of food samples for compliance with regulations regarding maximum permissible levels of synthetic colors including AR. To begin with, this technique offers numerous advantages over other techniques used today. One major advantage lies in its speed, great selectivity, green method, and analysis of AR without any pretreatment of the samples as compared to conventional methods such as spectrophotometry [4–8]. The use of N@CQDs allows for real-time results making monitoring more efficient and effective. Additionally, this approach ensures greater sensitivity and accuracy since nitrogen-doped quantum dots have unique optical properties making them highly responsive to changes in molecular structure [16, 17].

The researchers’ focus has recently shifted more toward carbon quantum dots (CQDs), which differ from their bulk counterparts in terms of size-dependent characteristics [17–19]. Carbon quantum dots are a new class of fluorescent nanomaterials that possess the attractive properties of high stability, good conductivity, low toxicity, environmental friendliness, and simple synthetic method. CQDs have significant applications in biomedical imaging, optoelectronic devices, electrochemical reduction reactions, sensors, pharmaceuticals, the food industry, and labeling due to their chemical and physical characteristics [20]. However, the organic-based methods are more complex, use more expensive and poisonous precursors, enhance environmental pollution, and operate at higher temperatures than aqueous-based approaches [19, 21, 22].

In this article, we present a simple, selective, and effective method for producing nitrogen-doped carbon dots for use in quantification of AR in food samples.

## Experiments

### Chemicals and Reagents

The chemicals as AR (99.70%) were obtained from Alpha Chemical (India). Sucrose, urea, methanol, ethanol, acetonitrile, and acetone were obtained from EL-Nasr Co, for pharmaceutics (Egypt). From the local market, various samples of beverages (orange soda, pink grapefruit soda, sports drinks) were collected (Egypt) and used directly without pretreatment.

AR stock solution (50 µg mL^− 1^) was made by dissolving 5.0 milligrams in 100 mL of double distilled water.

## Instrumentation

The presented work was performed via various equipment, FS5 fluorometer (UK) with a 150 W xenon lamp source for excitation. High-resolution transmission electron microscope (HR-TEM) images were captured via a JEOL JEM-100CX II unit with tungsten EM filament 120 (USA). The dynamic light scattering measurements (DLS) were scanned by the Zetasizer Red badge instrument of ZEN 3600 (Malvern, UK). MFMI-100 A (MED Future) microwave instrument (2450 MHZ 1000 W) was designed for catalyzing organic synthesis and solvent extraction. The powder X-ray diffractometer (PXRD) was scanned by a Philips X-ray diffractometer. Fourier transforms infrared (FT-IR) for N@CQDs was reported in a Nicolet™ iS™10 FTIR spectrometer in the wave number range 400–4000 cm^− 1^.

## Synthesis of Nitrogen-Doped Quantum Dots (N@CQDs)

N@CQDs were synthesized with a microwave-assisted method [16, 17], 0.5 g of sucrose and urea were dissolved in 50 mL of ultrapure water. The result was transferred to a 125 mL glass round bottom flask with a lid and then implanted in the microwave. Heat at microwave power (700 W) 2450 MHz for 8 min until a brown result forms. The residue was diluted with 50 mL of ultrapure water and then sonicated for 20 min to remove large particles. The mixture was filtered through a 0.45 μm membrane and centrifuged at 5000 rpm for 15 min. After that, the supernatant was filtered through a 0.45 μm membrane. The yellow solution was lyophilized for N@CQDs characterization.

## General Analysis Procedure

Various concentrations of standard solutions of AR were mixed with 1.0 mL N@CQDs (2 mg/mL) into 5-mL volumetric flasks. The solution was mixed with 1.0 mL BR buffer (pH 3.2) and completed to the mark with ultrapure water. Then the luminesces were measured after 5 min at 445 nm (excitation at 350 nm).

## Results and Discussion

**Fig. 1:**
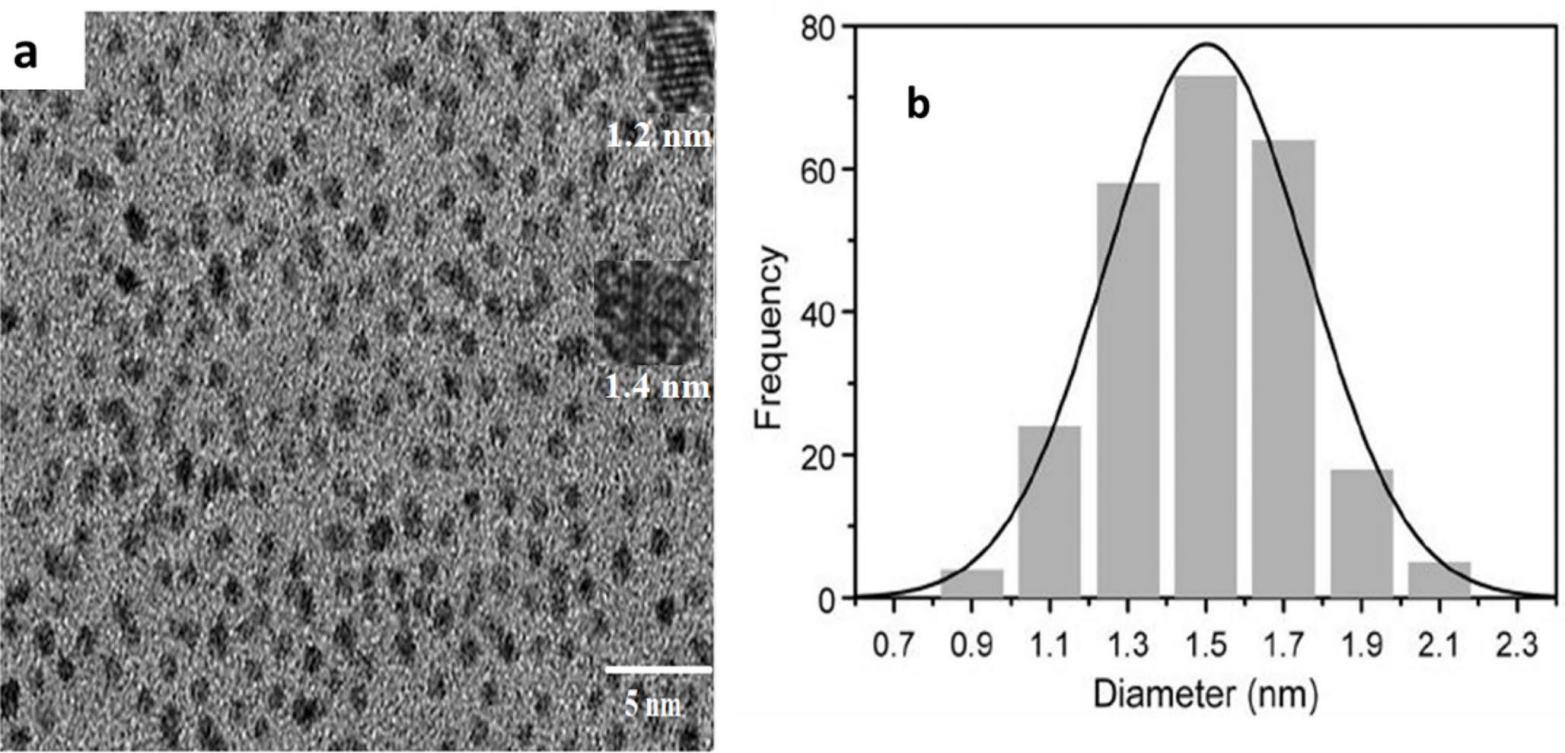
TEM image for nitrogen-doped carbon quantum dots and **b)** dynamic light scattering curve for the particle size of the quantum dots

The surface characters of the nitrogen-doped quantum dots (N@CQDs) were characterized using a TEM image of the green synthetical N@CQDs showing dispersed nanoparticles with regular diameters ranging from 1 to 1.6 nm (Fig. 1a**).** The size distribution in Fig. 1b displays that the average hydrodynamic diameter of N@CQDs is ~ 1.5 nm attained from the DLS result which agrees with the TEM image.

**Fig. 2:**
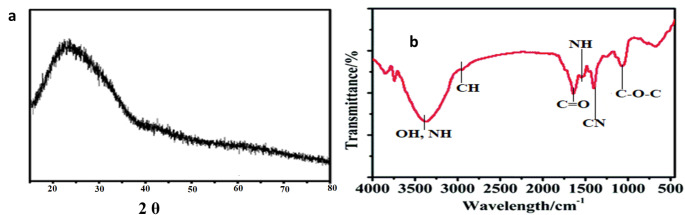
PXRD curve for the quantum dots, and **b)** FTIR spectroscopy for N@CQDs

The PXRD data from the point show strong diffraction peaks at ~ 24.30° (002) pattern (Fig. 2a). Demonstrating the amorphous description of synthesized N@CQDs [17, 18, 23]. The functional groups on the surface of the N@CQDs were investigated by FTIR spectroscopy (Fig. 2b), showing distinct bands at 3430 cm^− 1^ interrelated to the stretching vibrations of -NH and -OH groups, respectively. The bands at 2950 cm^− 1^ belong to the stretching and bending modes of single bond -C-H. The FTIR bands at 1223 cm^− 1^ correspond to the C-O-C bond stretch, in addition to the diagnostic peak at 1700 cm^− 1^ assigned to the -C = O which indicates the presence of -COOH in synthesized quantum dots. The unique bands at 1490 and 1326 cm^− 1^ associated with the -NH and -CN stretches indicate the presence of abundant nitrogen in the C-dot framework [24]. Figure S2 displays the EDX investigation for elemental analysis of the N@CQDs with detectable peaks corresponding to the elements C, N, and O. It was observed that the percentage of elements is 53.60%, 25.79%, and 20.61 for C, O, and N respectively.

XPS was carried out for elemental analysis of N@CQDs, the results of the XPS spectrum (Figure S3a) have shown that there are four different characteristic peaks near 285.10, 399.86 and 531.62 eV, which belong to the C1s, N1s, and O1s, respectively. For C 1s (Figure S3b), four peaks wear observed for C-C at 284.23 eV, C = C at 284.50 eV, C-N at 285.88 eV, and C = O at 287.85 eV. The O1s spectrum as shown in Figure S3c displays three peaks corresponding to C = O 531.22 at eV, C-O-H at 531.85 eV and O-C-O at 533.40 eV, respectively. The N1s scan spectrum (Figure S3d) has two characteristic peaks at 399.11 and 400.10 eV, which related to N-H and C-N-C, respectively [16, 24].

The quantum yield QY for N@CQDs was estimated using quinine sulfate as standard,

$${\varvec{Q}}_{\varvec{x} }= {\varvec{Q}}_{\varvec{s}\varvec{t}}\bullet \frac{{\varvec{I}}_{\varvec{x}}}{{\varvec{I}}_{\varvec{R}}}\bullet \frac{{\varvec{A}}_{\varvec{s}\varvec{t}}}{{\varvec{A}}_{\varvec{x}}}\bullet \frac{{\varvec{?}}_{\varvec{x}}^{2}}{{\varvec{?}}_{\varvec{s}\varvec{t}}^{2}}$$

The mean of QY of the four N, C-dots was calculated to be 36.60%.

### The Optical Performance of N@CQDs

Nitrogen-doped carbon quantum dots (N@CQDs) are nanoscale particles that exhibit unique optical properties such as fluorescence and UV absorption. These optical characteristics make them ideal for detecting Allura red in food samples. The fluorescence of quantum dots allows for sensitive detection of Allura red at low concentrations. Additionally, their UV characters enable precise quantification of the amount of Allura red present in a sample.

**Fig. 3:**
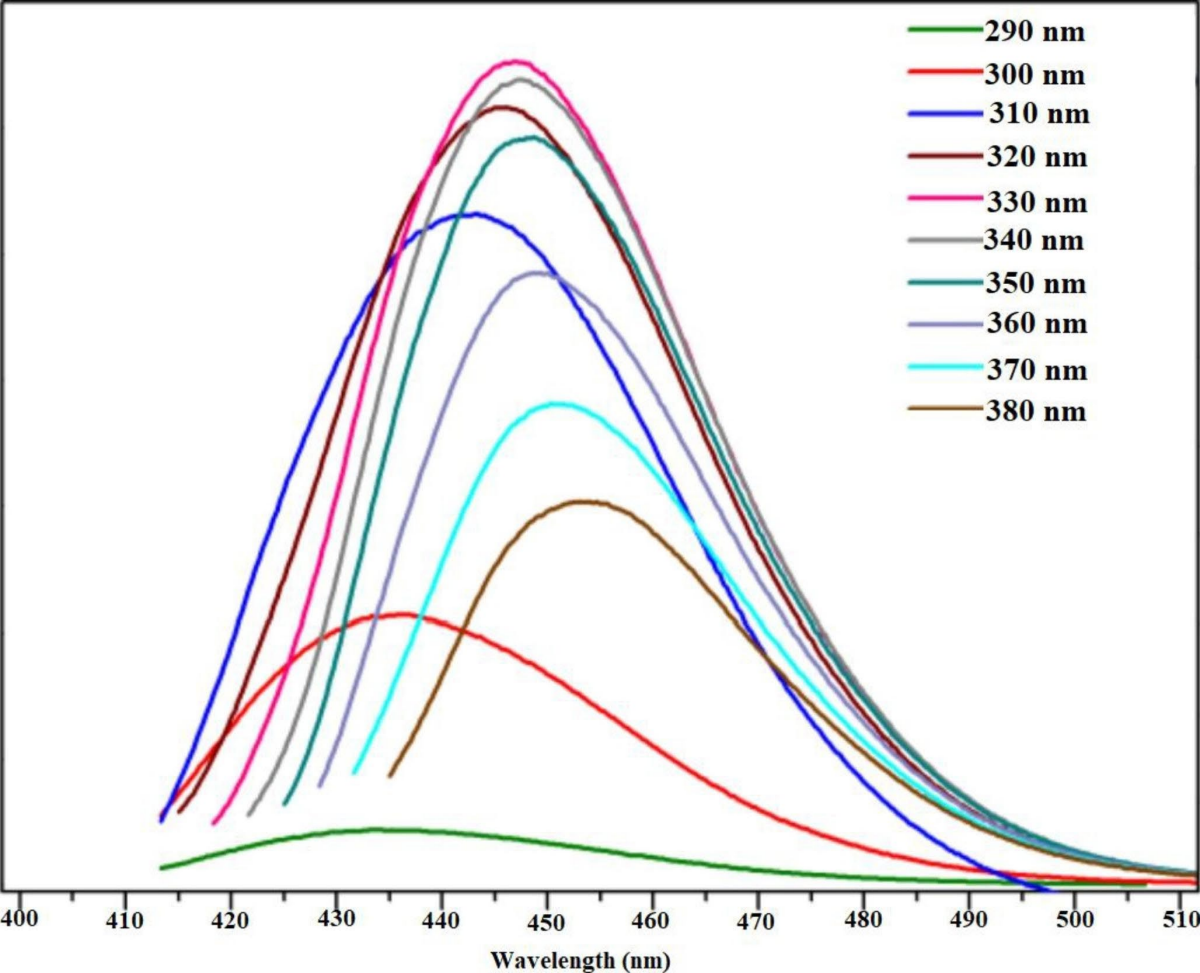
Excitation dependent emission spectra for N@CQDs

Figure S4 displays the UV characterization of the N@CQDs which provide two wavelengths at 253 and 335 nm [17, 25, 26]. On the other hand, different excitation wavelengths were applied for excitation dependent process. As found in Fig. 3, redshifts for the fluorescence curves were observed using different excitations which proves the excitation-dependent emission effect [17, 18, 24].

### Mechanism Performance of the Reaction

**Fig. 4:**
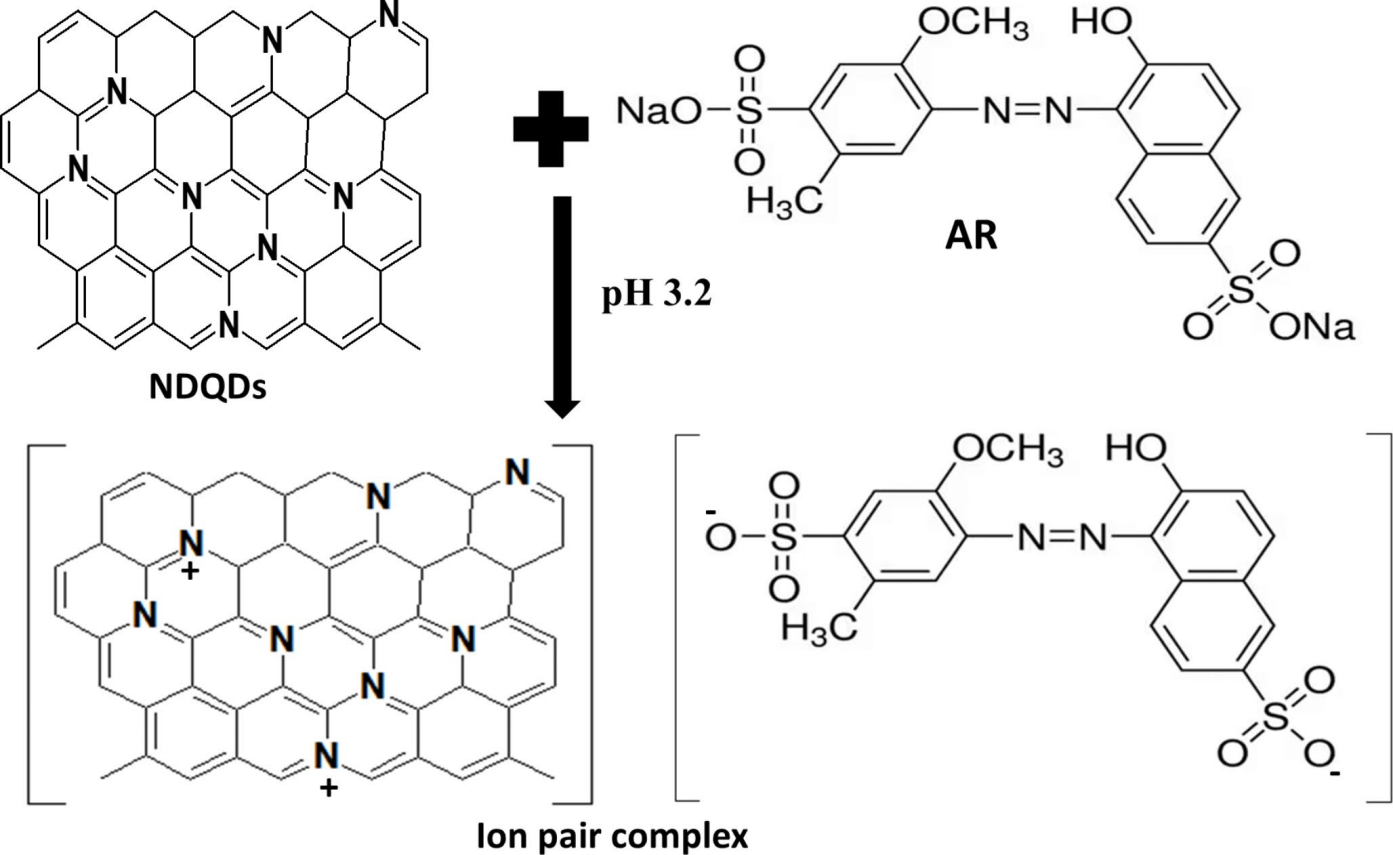
Ion pair complex between the nitrogen-doped carbon quantum dots and AR

As seen in Fig. 4, N@CQDs are protonated at pH 3.2 because they have a nitrogen atoms in their carbon skeleton that gives the probe basicity. AR, on the other hand, is an anionic hydrocarbon domain with negatively charged sulfonic acid moieties, conditional negative sites to form ion-pair complexes by electrostatic attraction with the positively charged N@CQDs [23, 27, 28]. The absorption spectra were shifted to redshift at about 24 nm (Figure S4) of N@CQDs upon exposure to AR indicating that the aggregation of N@CQDs is due to the interaction between the negatively charged sulfonate anion of AR and the positive charge of AR which confirm the ion pair mechanism [23]. To confirm that the binding between N@CQDs dots and AR interaction, the zeta potentials of N@CQDs, AR, and the complex was carried out (Figure S5). The zeta potential of the obtained N@CQDs was + 21.33 mV and AR -15.12 mV. After the ion pair complex formation, it was observed shifting the positive value of the zeta potential of the N@CQDs points to a less positive value after AR incorporation to + 5.80 mV which indicates the complex formation between quantum dots and Allura red.

In addition, an undoped quantum was synthesized to confirm the reaction mechanism of sulfonate anion in AR with nitrogen in the quantum dots. It was observed no significant interaction between Allura red (3.0 µg mL^-1^) and undoped quantum dots as shown in Figure S6.

**Fig. 5:**
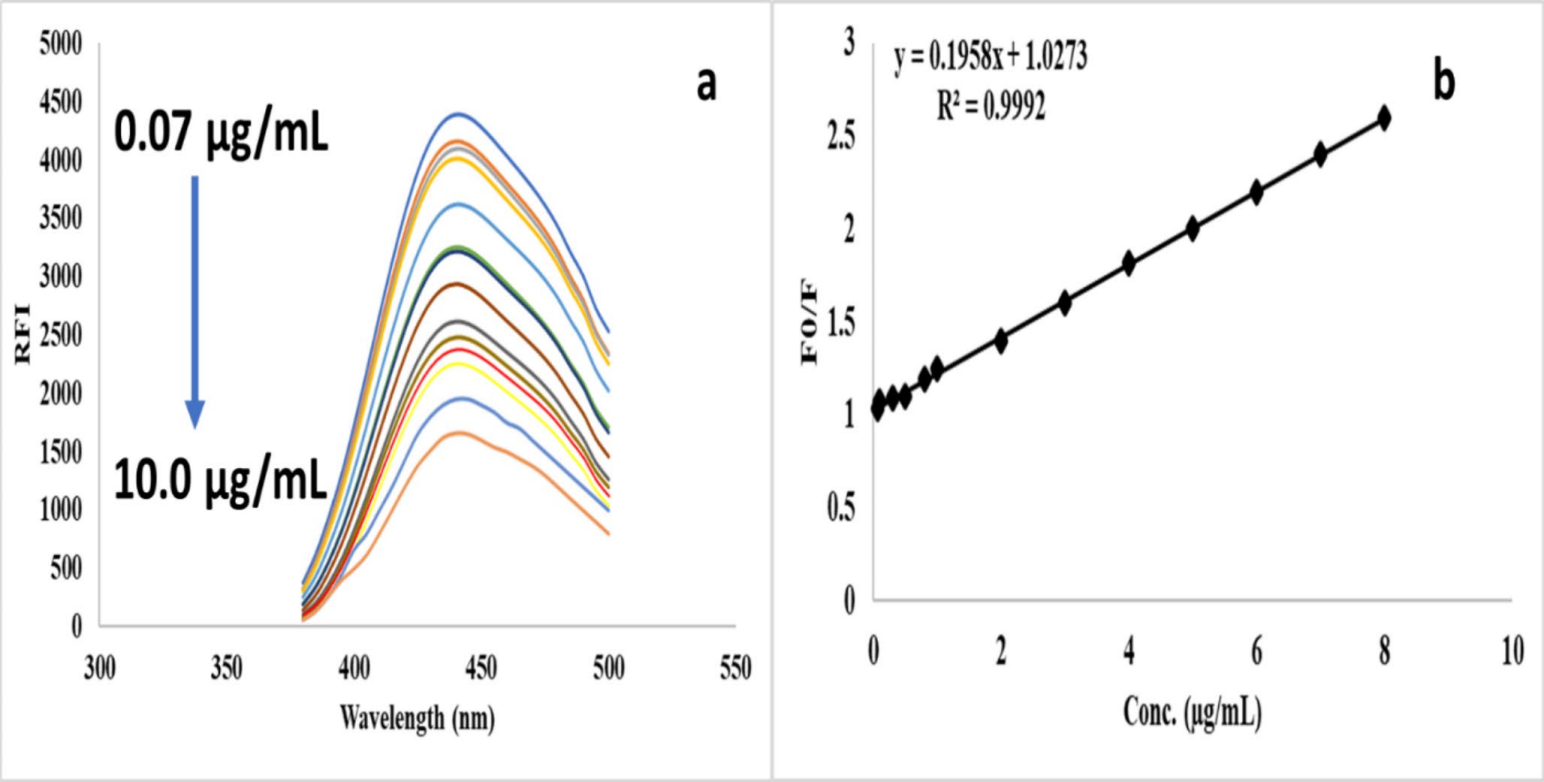
**a)** Reaction of N@CQDs with various concentrations of AR, and **b)** Stern-Volmer graph for the proposed method

The reaction of N@CQDs and AR was carried out at 440 nm after excitation at 350 nm as shown in Fig. 5a.

The reaction mechanism between AR and N@CQDs was also interpretated and confirmed with Stern-Volmer equation as:

F_0_/F = 1 + Ksv [Q].

The linearity of Stern-Volmer plot is a clear indication on the dynamic quenching mechanism. AR interacts with the excited N@CQDs resulting in ion pair reaction and led to quenching of the fluorescence of the quantum dots. This process is exactly described by the Stern-Volmer model. Figure 5b.

### Analytical Condition Optimization

In this work, we explore recent developments in the field of reaction optimization between N@CQDs and Allura Red. We delve into how different factors such as temperature, pH, and concentration affect the outcome of this reaction.

**Fig. 6:**
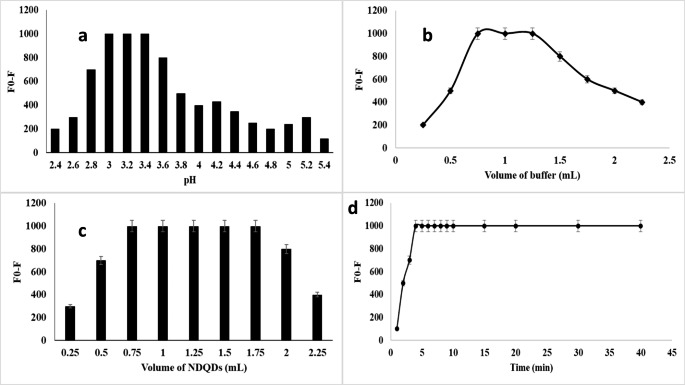
**(a)** Effect of pH, **(b)** Effect of volume of the buffer, **(c)** Effect of Volume of N@CQDs, and **(d)** Effect of the reaction time on the RFI using AR (1.0 µg mL^− 1^)

The effect of pH range on the fluorescence intensity was studied in the range of 2.4 to 5.4. It was observed that the acidic medium at pH (3.2) was the most suitable medium for the interaction between AR and N@CQDs, so pH 3.2 was selected as the optimum pH (Fig. 6a) using 1.0 ± 0.25 mL as in Fig. 6b.

The effect of volume of N@CQDs on the luminance reaction between N@CQDs and AR was observed that 1.0 ± 0.25 produce the stable quenching effect to the fluorescence intensity. Figure 6c.

The investigation of the effect of time response on the fluorescence intensity was settled that, the maximum time effect was observed after 5 min and no significant effect appreciably up to 40 min. Figure 6d.

### The Validation Performance of the Reaction

The presented study was validated according to ICH recommendations [29] as linearity, LOQ, LOD, accuracy, precision, and robustness were performed.

Figure 5a displays the linearity of the reaction of AR with N@CQDs found in the range of 0.07 to 10 µg mL^-1^ the reaction color was observed in Figure S7. The results were plotted on a graph using the Stern-Volmer Eqs.  [18, 19]:

F_0_/F = 1 + Ksv[Q].

as seen in Fig. 5b. The resulting linearity was evaluated by calculating the correlation coefficient (r^2^). The linearity was found in an acceptable range with r^2^ equal to 0.9992. Table 1.

**Table 1:** Statistical analytical parameters for analysis of AR

Parameter	Results
**λ**_**ex**_ **(nm)**	350
**λ**_**em**_ **(nm)**	445
**Concentration range (µg mL** ^**− 1**^ **)**	0.07 − 10.0
**Determination coefficient (r** ^**2**^ **)**	0.9992
**Slope**	0.195
**Intercept**	1.02
**SD the intercept (Sa)**	0.001
**LOD (µg mL** ^**− 1**^ **)**	0.01
**LOQ (µg mL** ^**− 1**^ **)**	0.05

Limit of detection (LOD) and limit of quantitation (LOQ): LOD and LOQ are important parameters to determine the sensitivity of the analytical method. The LOD and LOQ values for the creative method were found to be 0.01 and 0.05 µg mL^-1^, respectively. These values are within the acceptable range and indicate that the method is sensitive enough for its intended use. Table 1.

Known concentrations of AR (1, 2, 3, 4, and 5 µg mL^-1^) were prepared and analyzed using the quantitative analytical method to study the accuracy. The percentage recovery of the method should be within an acceptable range, typically defined as 95–105% [29]. In this case, the percentage recovery was found to be 100.25 ± 0.84% to 102.50 ± 0.55%, which is within the acceptable range. Therefore, the results refer to the high accuracy of the N@CQDs method. Table 2.

**Table 2:** Estimation of accuracy and precision of selected approach

Sample number	Taken Conc. (µg mL^− 1^)	Found Conc. (µg mL^− 1^)	% Recovery ^*^ ± RSD
**1**	1.0	1.01	101.00 ± 0.60
**2**	2.0	2.05	102.50 ± 0.55
**3**	3.0	3.06	102.00 ± 0.21
**4**	4.0	4.01	100.25 ± 0.84
**5**	5.0	5.10	102.00 ± 0.81
**Intra-day** **precision**	2.0	2.03	101.50 ± 0.77
	4.0	4.0.2	100.50 ± 0.51
	6.0	6.06	101. 00 ± 0.94
**Inter-day** **precision**	2.0	2.01	100.50 ± 0.70
	4.0	3.99	99.75 ± 0.80
	6.0	5.94	99.00 ± 0.35

^*^: Average of three determinations. **RSD**: Relative standard deviation

The precision of the proposed method was studied using concentrations of AR (2, 4, and 6 µg mL^-1^) and analyzed using the quantitative analytical method. The relative standard deviation (RSD) of the results should be within an acceptable range, typically defined as less than 5%. In the presented work, the RSD was found to be 0.35–0.94%, which refers to the high repeatability and reproducibility of the analytical method. Table 2.

Robustness: The analytical method could be intentionally varied by changing one or more experimental parameters, such as pH, the volume of buffer, the volume of N@CQDs, and the time of the reaction. The results refer to no significant effect of a minor change in analytical procedure as seen in Table 3.

**Table 3:** Robustness of the nitrogen-doped carbon quantum dots reaction with AR (2.0 µ mL^− 1^)

Variations				
		% Recovery ^a^ ± RSD		
**Optimum condition**		102.11 ± 0.40		
**1- Effect of pH (BR buffer)**				
3.0		100. 10 ± 0.41		
3.4		99.98 ± 0.29		
**2- Volume of buffer (mL)**				
0.75		99.50 ± 0.53		
1.25		99.79 ± 0.82		
**3- Cu-NDQDs concentration (mg mL** ^**− 1**^ **)**				
1.75		99.49 ± 0.66		
2.25		99.81 ± 0.50		
**4- Reaction time (min)**				
4		99.90 ± 0.59		
6		100.11 ± 0.30		

^*^: Mean of three determinations

### The Selectivity of the Proposed Method

**Fig. 7:**
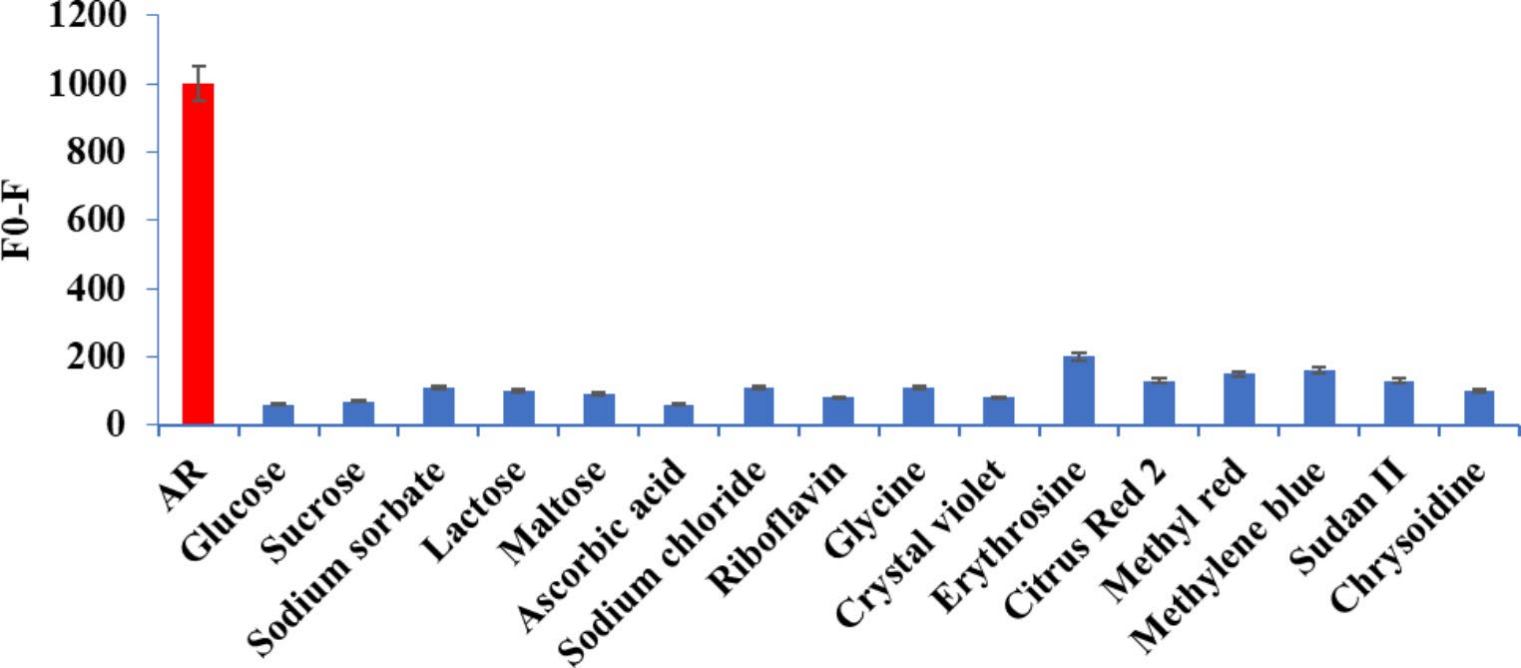
Selectivity study of AR (1.0 µg mL^− 1^) with N@CQDs comparing with other ingredients

To examine the selectivity of the suggested reaction between N@CQDs and AR was carried out by specific compounds such as glucose, sucrose, lactose, maltose, sodium sorbate, ascorbic acid, riboflavin, glycine, crystal violet, erythrosine, citrus red 2, methyl red, methylene blue, Sudan II, and chrysoidine (1.0 µg mL^-1^). As seen in Fig. 7, no significant interfering effect from the studied compound with the fluorescence intensity of N@CQDs. The results refer to the high selectivity of the proposed method.

### Quantification of AR in Beverages Using N@CQDs

In last years, the fluorimetric approach is the most applicable technique for the analysis of different products due to its simplicity, sensitivity, coast-effective, speed, applications in clinical laboratories and it’s selectivity [30, 31]. In this work, the beverage samples (orange soda, pink grapefruit soda, and sports drinks) were effectively analyzed directly without any pretreatment using N@CQDs. Overall, the use of N@CQDs for the quantification of AR in beverages is a promising area of research that has the potential to provide a more accurate and efficient method for food safety and quality control. The results were calculated and presented in Table 4 and agree with other reported methods [32, 33].

**Table 4:** Application of the N@CQDs method for quantification of AR in beverages

Samples	Added conc. (µg mL^− 1^)	Found Conc. (µg mL^− 1^)	% Recovery ^*^± RSD
**Orange soda**	0.0	7.80	100.40 **± 1.30**
	1.0	8.90	114.10 **± 0.58**
	1.5	9.00	115.38 **± 0.97**
**Pink grapefruit soda**	0.0	1.80	100.32 **± 0.25**
	1.0	2.29	127.22 **± 1.11**
	1.5	2.80	155.50 **± 1.80**
**Sport drink (Mountain DEW)**	0.0	5.54	100.60 **± 0.73**
	1.0	6.50	118.18 **± 0.91**
	1.5	7.00	126.35 **± 1.05**
**Sport drink** **(Liliac)**	0.0	2.89	100.07 **± 0.77**
	1.0	3.90	134.94 **± 1.55**
	1.5	4.00	138.40 **± 1.95**

^*^: Average of three determinations

## Conclusion

In conclusion, the determination of AR using N@CQDs is a revolutionary method in the food industry. AR is widely used as a color additive and it’s important to ensure its safety for human consumption. The use of nitrogen-doped carbon quantum dots offers several advantages such as high sensitivity, specificity, great selectivity, and fast detection time. This technique has brought about a paradigm shift in the analysis of food additives. With this method, we can detect even minute quantities of AR with ease, making sure that our food is safe for consumption. It’s truly amazing how technology has revolutionized the way we test our food for safety purposes. In short, N@CQDs have made life easier by providing us with an accurate tool to determine whether our food contains harmful chemicals like AR.

### Electronic Supplementary Material

Below is the link to the electronic supplementary material.

Supplementary Material 1

## Data Availability

All data generated or analyzed during this study are included in this published article (and its supplementary information files).
