# Supplementary material for: An Innovative Selective Fluorescence Sensor for Quantification of Hazardous Food Colorant Allura Red in Beverages Using Nitrogen-Doped Carbon Quantum Dots
Source: J Fluoresc. 2023 Jun 17;34(2):599–608. doi: 10.1007/s10895-023-03303-2 (PMC10914892; doi:10.1007/s10895-023-03303-2)
Supplement: Supplementary file 1 — Supplementary Material 1 [file 10895_2023_3303_MOESM1_ESM.docx]

**An Innovative Selective Fluorescence Sensor for Quantification of Hazardous Food Colorant Allura Red in Beverages Using Nitrogen-Doped Carbon Quantum Dots**

**Baher I. Salman**

Pharmaceutical Analytical Chemistry Department, Faculty of Pharmacy, Al-Azhar University, Assiut branch, Assiut, 71524, Egypt.

[**bahersalman@azhar.edu.eg**](mailto:bahersalman@azhar.edu.eg)**,** [**bahersalman2013@yahoo.com**](mailto:bahersalman2013@yahoo.com)

**
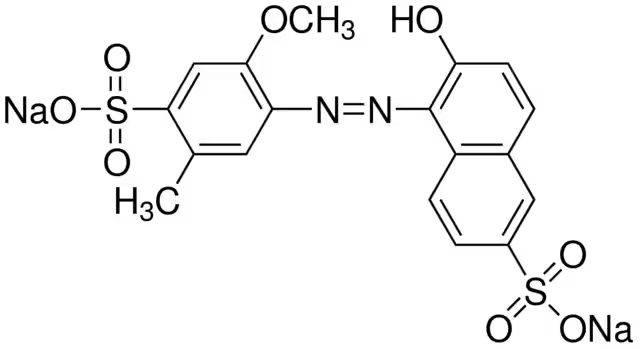
**

**Figure S1** Chemical structure of Allura red.


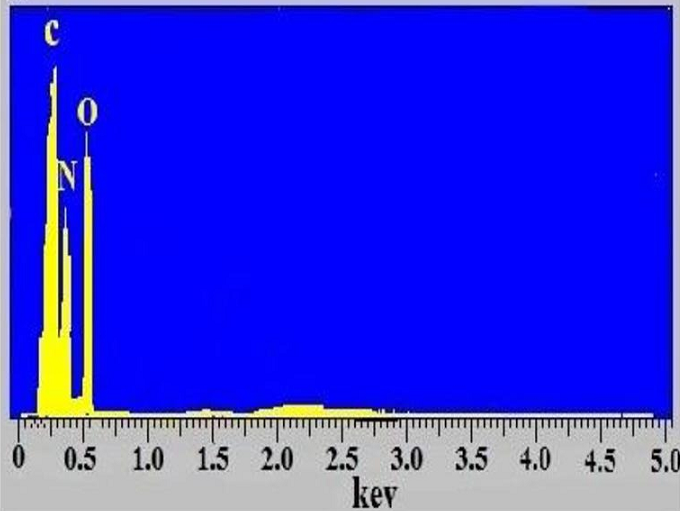


**Figure S2** Elemental analysis of the nitrogen-doped carbon quantum dots using EDX image.


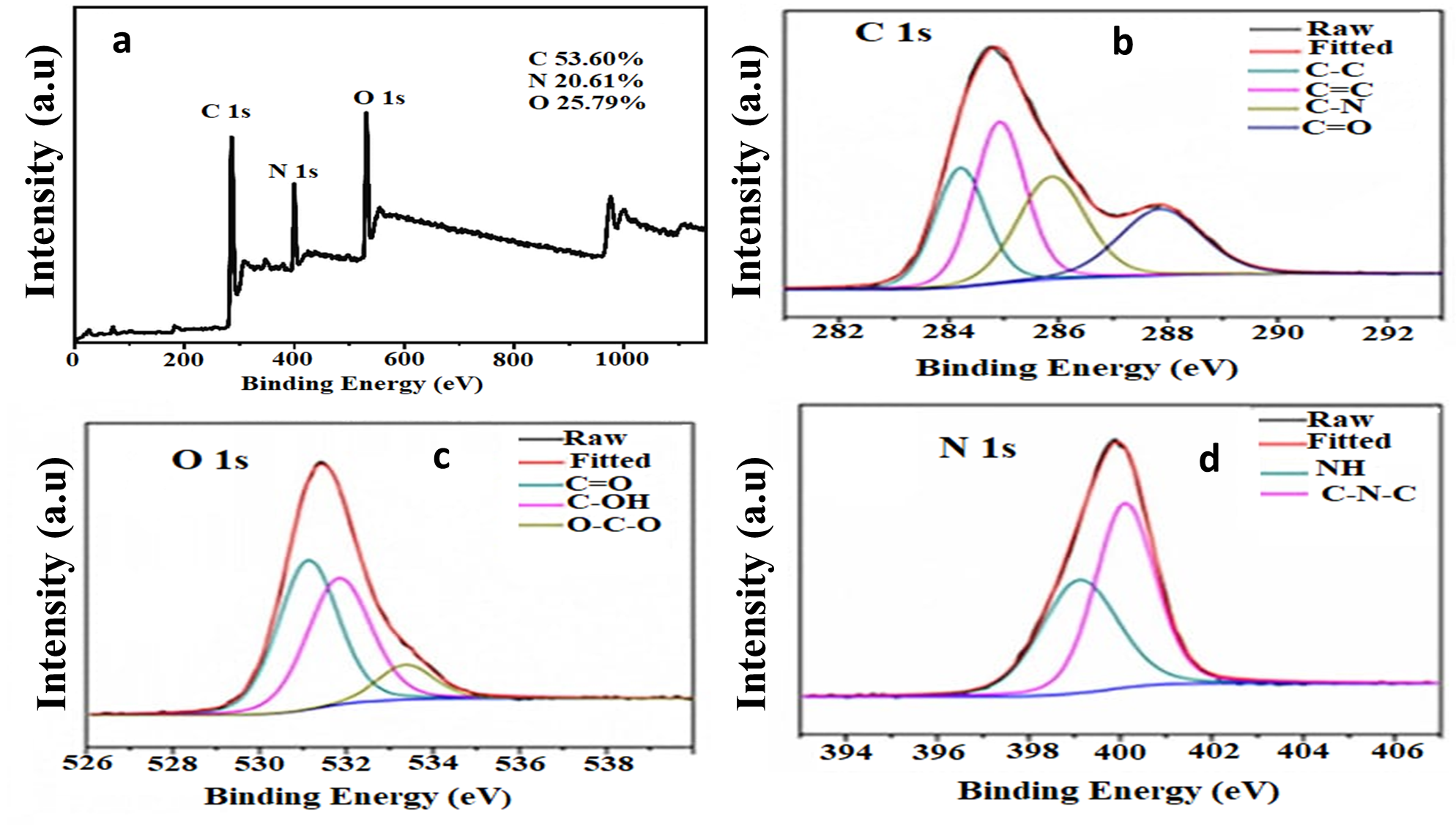


**Figure S3** Elemental analysis for N@CQDs, **a)** XPS spectra, **b)** C 1s spectra, **c)** O 1s spectra, and **d)** N 1s spectra.


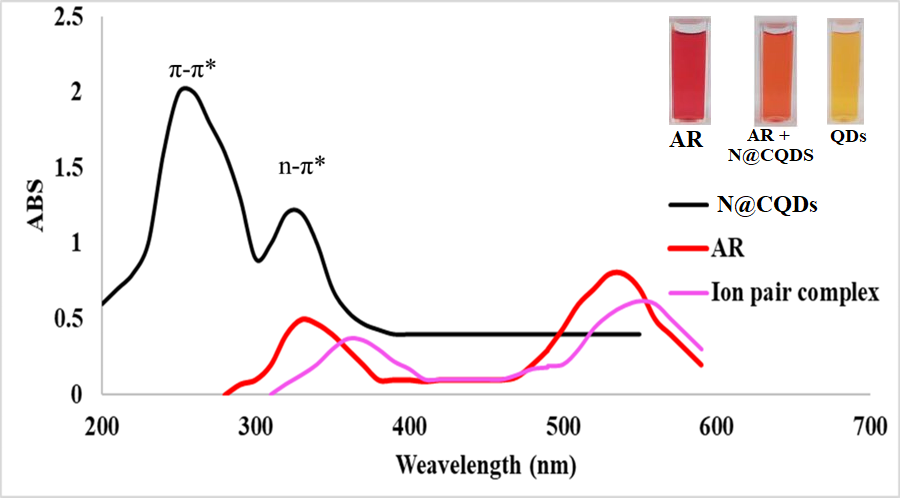


**Figure S4** UV optical characterization of N@CQDs.


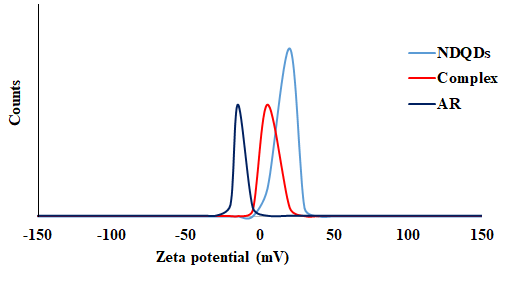


**Figure S5** Zeta potential of the quantum dots, AR and ion pair complex.


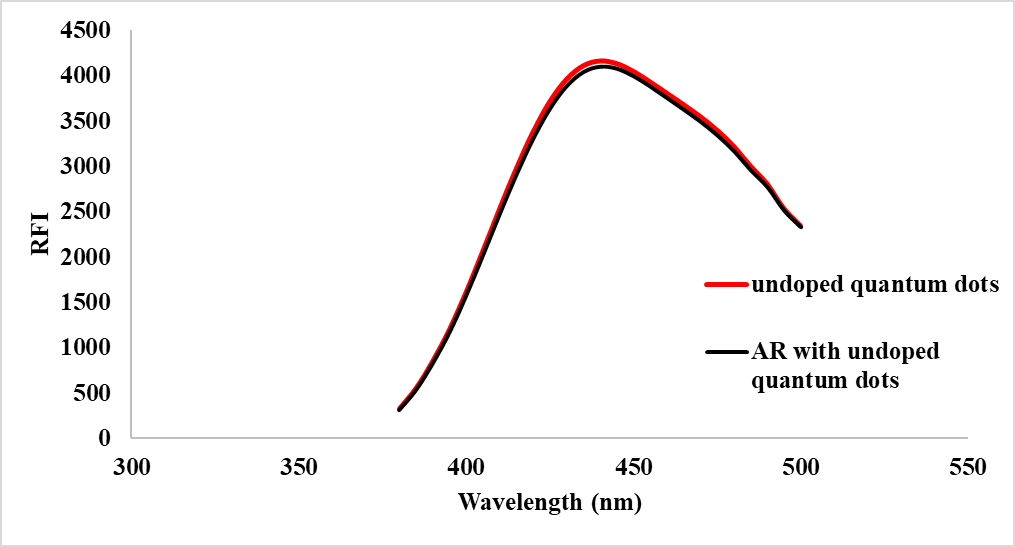


**Figure S6** Reaction of Allura red (3.0 µg mL^-1^) with undoped carbon quantum dots.


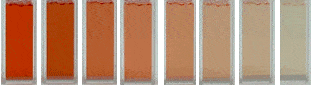


**Figure S7** Reaction of the N@CQDs with AR using different concentrations (0.07 – 10 µg mL^-1^)
